# Collective Emotions: A Case Study of South African Pride, Euphoria and Unity in the Context of the 2010 FIFA World Cup

**DOI:** 10.3389/fpsyg.2018.01252

**Published:** 2018-08-21

**Authors:** Gavin B. Sullivan

**Affiliations:** Centre for Trust, Peace and Social Relations, Coventry University, Coventry, United Kingdom

**Keywords:** collective emotions, group-based emotions, collective pride, crowd behavior, mega-sport events, FIFA Mens World Cup impact, social ontology, social identity theory

## Abstract

Collective emotions experienced as existing objectively and widely shared challenge traditional views of emotions based on personal or private interests. This paper extends theories of group and crowd emotions focusing on social appraisal, social identity, emotional contagion, and ecstatic nationalism, and adds an interdisciplinary approach to research on international mega-sporting event impacts and legacies by examining the national-level collective emotions produced by a mega-sport event—the 2010 World Cup in South Africa. The novel case study approach triangulates ethnographic observations of life in downtown Johannesburg before and during the World Cup with a critical thematic analysis of qualitative interviews of 10 South Africans and the author’s and publicly posted videorecordings of individual and collective behavior. I explore how citizen support for efforts to pursue national projects combined with international attention to generate widespread and genuinely coordinated collective emotions of euphoria and pride. The social ontology-based analysis considers bottom–up and top–down mechanisms of emotional spread and influence along with important expressive-performative contributions of culture-specific forms of group-based and collective action tendencies. Moreover, the study shows how group agency in the form of coordinated ritualistic bases realized group affects spontaneously and normatively as South Africans desired, accepted and celebrated achieving team and host-related group goals. These results provide new insights into the emotions that occur in public events in two phases, (1) creation of collective normative commitment in practice related to group ethos and national interests and goals prior to the tournament start, and (2) during the tournament when dynamic relations between group-based and collective emotions also generated feelings of unity and solidarity. Together they highlight unique predisposing cultural and historical features of the emotional and affective-discursive practices associated with the World Cup for South Africans, limits to the spread of emotions of enthusiasm from urban cities to rural areas, forms of excitement and celebration in public spaces, instances of ambivalence about efforts to enact support for the nation’s World Cup team and host role, and indicate how collective emotional experiences are internalized, embodied and reproduced in accounts of national transformation, concerns about fragile intergroup solidarity, and instances of group-based hubristic pride.

## Introduction

Collective emotions experienced as existing objectively “out there in the world” and widely shared in a given group challenge traditional views of emotions as private and reflecting personal interests (Salmela, 2014; Krueger, 2016). For citizens of many nations, emotions are regarded as experiences in which the body and the skin, particularly, is an important boundary; that is, first-person emotions may be described as similar to those of others but never as exactly the same. First-person plural expressions of emotion, in contrast, often capture a shared psychological experience such as when a team that one is associated with wins (e.g., “basking in reflected glory”; Cialdini et al., 1976). However, while the achievements of others can have a positive joint impact, analyzing statements like “we won!” can still imply an individualistic positive self-conscious emotion induced by others rather than one mutually amplified (Neville and Reicher, 2011) and shared by most members of a group. Smith et al.’s (2007, p. 431) theory of group-based emotions similarly focuses on individuals with a prior group connection who can be “elated when their favorite football team upsets a stronger team, saddened and angry when their team loses a game, and disgusted when the winning team’s fans drunkenly riot in the street—all without personally leaving the couch.” While in all these instances there can be fluctuations and variations in group identification within and between group members, focusing on a psychological process of identification is not sufficient to address the intensity of the feelings that can occur when a person joins with a crowd of loosely affiliated individuals (e.g., to watch a game) and is subsequently—due to the contingencies of the game—surprised to find themselves hugging, celebrating with and feeling bonded to people who were strangers just minutes before (Reicher, 2011). Moreover, because such collective emotions can occur at the level of nations, any investigation must be properly interdisciplinary and draw upon concepts and analyses developed across the social and psychological sciences and, where appropriate, the humanities.

Individually experienced and expressed group-based emotions are important to collective emotions as their dispositional precursors or internalized and embodied “after effects”; but the crucial difference is that collective emotions involve coordinated group-based affects in the actual or strongly implied co-presence of other people who focus on a common object of attention, want to see a particular group aim achieved and are prepared to work toward a shared goal (e.g., a significant political change effected by acting *as a group*; Collins, 2012). Accordingly, genuinely group-level affects incorporate and interact reciprocally with aggregate instances of emotions that individuals feel on behalf of their group or on the basis of group membership(s) (Sullivan, 2014a; Salmela and Sullivan, 2016; cf. Salice and Montes Sánchez, 2016). Furthermore, collective emotions are qualitatively distinct from the properties of sound, for instance, that emerge when a large group of people pursuing their own private ends is temporarily co-located in the same space. While the vocalized content and expressive qualities of a crowd are important in judging its emotion, more is involved than determining the crowd’s “average emotional expression” (McHugh et al., 2012). Thus, “group-based emotions consider an individual’s emotional experience in response to group-related events, whereas collective emotions refer to the collective as the entity that experiences [or better, exhibits] the emotion” (Goldenberg et al., 2014, p. 582, brackets added). Moreover, while social psychologists have investigated the “psychological group” (Reicher, 2011) it is important to focus on the conditions in which specific genuinely collective emotions occur (e.g., collective pride; Sullivan, 2015) as well as their dynamic relations to other emotions or affects and normative background conditions.

The philosophical literature on collective action emphasizes the intentionality of a group’s behavior (Tuomela, 2013) and the affective states such as feelings, emotions and sentiments that are generated in the pursuit of joint action (and can function as reasons to justify or motivate collective acts; Salmela and Nagatsu, 2016). The intentions shared in collective action are not reducible to anything located within individuals—including an influential leader—but rather “the joint goal is achieved *together* or… there is also a collective commitment to the goal as *our* goal” (p. 42). Such analyses may appear to also be beyond our everyday language about group emotions, but our everyday examples need not be subjected to radical revision. For example, while concepts such as “effervescence” appear to overlap with ordinary talk of feeling the excitement of a crowd “build,” the vocabularies used after experiencing a group positive outcome can be expressed in terms of elation, happiness and celebration but still reasonably be grouped together as cases of collective pride (Sullivan, 2014a). For instance, when a person states that “I’m happy for them” in a situation where group members celebrate and wave (or wear) national flags with many others, this can be treated as an instance of group pride. In this case study of the emotional impact of the FIFA 2010 World Cup in South Africa, therefore, conceptual and theoretical distinctions are important. For example, Drury and Reicher’s (2005, p. 35) investigation of the impact of political action on a protest group focuses on collective empowerment as a positive outcome which can endure beyond a given “victory” and is therefore “not reducible to the experience of success.” In contrast, Tausch and Becker (2013) discuss participants being “filled with pride” about achieving a positive outcome through participating in collective political action but their explanatory account does not address the practical conditions in which genuinely collective emotions occur as a result of prior collective commitment.

An important aim of the study reported below is to use social ontology analyses to determine why the emotional impact of the performance of the South African national team and the nation as a World Cup host is better described in terms of collective pride as well as unity and solidarity instead of collective empowerment. In addition, the investigation of what is “shared” does not only mean one individual expressing a largely private emotion to another person—even though communication and convergence of group-based emotions in conversations and related interaction rituals (Collins, 2004) is a useful model for understanding how emotions spread through social networks. Moreover, in what follows, it is accepted that collective emotions can be experienced—sometimes simultaneously—by people organized at different “levels” (e.g., individual, dyadic, organizational, *and* national; Barsade and Gibson, 1998; Ashkanasy, 2003) as a group attends to the same “object” or activity (Collins, 2004, 2012). A football World Cup is an appropriate focus because it is an international event in which intense group and group-based emotions reliably occur in virtue of being widespread among members of a national group (Ismer, 2011; cf. Kelly and Barsade, 2001 whose focus is only on work teams, small groups and organizations). Accordingly, theoretical issues and studies are critically reviewed beginning with the focus on relevant top–down and bottom–up generative processes before examining contrasting explanations from the social ontology literatures, exploring specific research on nationalism and collective emotions and then introducing accounts of the embodiment and enactment of group-based and collective emotions.

## Top–Down and Bottom–Up Collective Emotion Processes

A consistent theme in collective emotion research is to examine collective emotions by “moving from the group level downward (a top–down approach), as well as moving upward from the compositional effects of individual group member emotions (a bottom–up approach)” (Barsade and Gibson, 1998, p. 82). Top–down processes are argued to have the potential to homogenize, exaggerate and constrain individual emotions. Several significant features of group emotion are identified that include the individual experience of collective emotions as overwhelming and strong influences on thought and behavior that may still be resisted by one or more individuals for a variety of reasons (e.g., contrasting group allegiances, different personal values; Goldenberg et al., 2014). A further role of top-down processes highlighted by Barsade and Gibson (1998) is to constrain collective emotions; in particular, they argue that widespread norms often regulate group emotions rather than generate them spontaneously. However, more recent research indicates a wider range of influences on group affect such as group-level narratives and the emotions and actions of emotions of group leaders or representatives (e.g., in protest movements and nations; Jasper, 2014). Top–down approaches are described by von Scheve and Ismer (2013) as the contribution of culture and shared knowledge to the occurrence of cognitive emotions. Their general theoretical account acknowledges that group-level narratives are examples of representational formats that include mass media, art works and “cultural conceptions of what is usually felt or should be felt [that] are disseminated to large numbers of recipients, which in turn may promote the elicitation of collective emotions” and therefore allow collectives to “transcend individual cognition and behavior in generating symbolic and normative orders of meaning making” (von Scheve and Ismer, 2013, p. 411).

International sporting events also have the potential to shape the emotions of groups through collective memory which plays an important normative role within groups: “symbolic practices of remembering and commemoration and public discourse may establish society-wide conventions of what is remembered, in which ways, and with which emotional consequences” (von Scheve and Ismer, 2013, p. 411). Focusing on the 2006 World Cup in Germany, Sullivan (2009, 2014b) analyzed media event rituals and the multiple interacting top–down and bottom–up processes normatively constraining expressions of national pride to understand how a transformation of collective shame occurred. In addition, banal unreflected upon uses of “us” and “we” (Billig, 1995) inform the group-based and collective emotions that are possible about one’s nation along with practices and narratives of the desirability (or otherwise) of group pride (e.g., downplaying the relevance of signing a national anthem, rituals that reinforce the importance of national symbols such as flags, etc.). While quotidian conversational practices and rituals constitute bottom–up processes, von Scheve and Ismer (2013) note that theoretical explanations should include mechanisms of face to face contact and mimicry, group identification, and emotional contagion. As we will see below, face-to-face contact does not address issues such as the coordinated sounds that crowds can create when acting as a group (cf. McHugh et al., 2012), mimicry explanations should address how normative pressure works to align people’s seemingly spontaneous emotional expressions, identification with a group is not sufficient to explain group dispositions and norms that emerge from participation in shared nation-related discursive practices, and emotional contagion theories often ignore features of voluntary behavior (e.g., choosing and controlling the sharing of emotions verbally and physically with others) thereby risking the reintroduction of myths of “mob” identity dissolution, irrationality and loss of control. However, an explanatory focus on causal mechanisms and methodological individualism omits important features of group agency highlighted in social ontology analyses that are not addressed in social identity accounts of crowd collective emotions (e.g., Neville and Reicher, 2011) and does not address the dynamic interplay between specific emotions (e.g., excitement, euphoria, pride) and changing experiences of unity, relatedness, togetherness, belonging and solidarity.

### The Contribution of an “I-Mode” and “We-Mode” Social Ontology Analysis

A promising development in recent analyses of collective emotions is the contribution of scientifically testable social ontology framework (Tuomela, 2013). Salmela (2014) highlights the central distinction between “I-mode” and “we-mode” collective motivational and emotional behavior, intentions and action. Here “I-mode” means that an individual engages in group behavior primarily in terms of private reasons or personal interests. An individual may therefore be present in a crowd in prosocial “I-mode” but because their enjoyment is mainly personal (or because they experience physical discomfort; cf. Novelli et al., 2013), they will not experience “we-mode” collectivity. Tuomela (2013) and others accept that collective emotions usually involve cognitive shifts and orientations toward a shared or social identity but note that this can be conceived individualistically (in terms of pro-group I-mode states and activities) and not as the “full-blown collective social identity of persons consisting of their we-mode states and activities (that are conceptually group-dependent)” (p. 12). Such changes can reflect cognitive appraisals and social appraisals that people have in common which generate “I-mode” collectivity (von Scheve and Ismer, 2013). For example, a citizen who sees everyone dressed in national colors may not only feel positive about them and experience a sense of belonging to the group but may also think “I should support the team” and “the World Cup is an important event”. However, even when a national team wins that same citizen may only experience happiness or something like personal pride (e.g., “I’m proud that I supported the team”) rather than a genuinely shared and collective pride (Sullivan, 2014a). In such cases, social identity and categorisation theorists emphasize the importance of the “cognitive transformation” (Hopkins et al., 2016, p. 22) of individuals in a crowd orienting toward group norms, but social ontology accounts include the irreducible collective consequences of individuals in a group acting together in we-mode form, the objectively real dispositional features of a group acting in we-mode and the stronger functionality of their collective emotions (i.e., than an I-mode group).

A further requirement for collective emotions to occur in a group (in contrast to emotions based on group membership which can be individualistic and idiosyncratic as indicated by some instances of national pride) is collective commitment to the ethos of the group (i.e., to its constitutive interests, goals and principles). As Tuomela puts it, such emotions “may also be based on shared individual emotions induced by the group, but the emotions still might not be fully collective in the sense of having been collectively accepted by the members” (p. 250). The analysis is appropriate for the case study of South Africa because it can be used to account for the collective emotions and actions of citizens organized as a national group. The account generates the testable hypothesis that the “justificatory direction in the we-mode is ‘top–down’ from group-level to member level, whereas in the I-mode the primary conceptual and justificatory as well as ontological direction is ‘bottom–up,’ from member level to group-level” (p. 5). It also extends Kelly and Barsade’s (2001) speculations about the ways in which small group emotion can feed back into the affective antecedents of group emotion to the national level after considering also the interrelationships between affective and non-affective factors (e.g., the impact of intergroup relations, physical spaces and communicative technologies). It is therefore important to examine empirically instances of collective emotion and details of (1) any reference to group reasons being authoritative (e.g., about the reasons why South Africa should host the World Cup, including why supporting the team is important and how relevant actions advance group interests in accordance with a shared group ethos), (2) how collective commitment is formed in group practices and actions, (3) evidence of the time-course and dynamic nature of collective emotion which are not reducible to individual’s personal and group-based emotional experiences, and (4) situations where collective action tendencies are realized in ways that are recounted explicitly by participants in terms of feelings of group pride, national unity and solidarity.

Accordingly, the social ontology framework incorporates how “the group constitutes the social identity of each individual ‘we-moder”’ (Tuomela, 2013, p. 24) but focuses also on three features that are not present in social identity theories: group reasons, collectivity (i.e., acceptance that group members are in the same boat), and collective commitment to the satisfaction of the group’s goals. It is not possible here to outline fully how examining these features overlaps with or extends social identity theories, but they do indicate why people feel uncomfortable acting in accordance with their private interests once they have experienced genuinely collective positive experiences. This is not only because “group pride motivates people to approach other ingroup members or to increase their level of identification with the group” (Smith et al., 2007, p. 433): people also require the permission of the group to disengage from a project to which they jointly (and freely) committed. Social identity theorists focus on identity content, identity boundaries and highlight close links between collective actions and group-based feelings which can converge toward a group prototypical form (Smith et al., 2007; Ray et al., 2014), but there is little role for genuinely collective “we-mode” emotions and their group-level prerequisites and consequences. Moreover, social identity theorists exclude neo-Durkheimian approaches focusing on ritual, effervescence and emotional entrainment (e.g., Collins, 2004, 2012; von Scheve et al., 2014; cf. Hopkins et al., 2016), usually emphasize instead the implications of emotions for subsequent collective actions or commitment (Neville and Reicher, 2011) and can be highly critical of any account of collective emotion relying on emotional contagion where this implies crowd irrationality and loss of identity (cf. Sullivan, 2015).

### Research on Mega-Sporting Events, Collective Emotions and National Identity

Relevant theories and studies of collective emotions are also found in studies of mega-and other sporting events which include their connections with nationalism, patriotism and national identity. Collins (2004), for instance, analyzed football crowds but suggested that their rituals were more important in generating collective emotions than the outcome game. Accordingly, the approach might better explain solidarity and collective commitment among long-term supporters than people following an international football tournament. Cottingham (2012), in contrast, suggests that Collins’ account of sports fans “implies weak solidarity” (p. 171) and appears to conform with what Tuomela (2013) calls “I-mode groups” which have comparatively weaker solidarity between group members. Moreover, despite focusing on specific fans of a United States football team rather than on citizens of a host nation of a mega-sport event, Cottingham’s study indicates how peak ritual experiences of “emotional energy” might encourage relative newcomers to very quickly regard themselves as supporters of a given team and it helps to understand why these are reported in terms of specific emotional categories of group joy, happiness, pride, solidarity or unity.

Mega-sporting events and international sporting “carnivals” are a key means by which “banal” forms of nationalism are transformed temporarily into moments of “ecstatic nationalism” (Skey, 2006). But support for national teams in international football tournaments does not reflect a straightforward relationship with a more fundamental national patriotism (e.g., see Abell et al.’s. (2007) research with supporters of England). Mega-sport events have considerable potential to generate positive collective emotions such as national pride in nations through a combination of factors such as: the extent of citizen identification with national representatives and teams, the manner of individual and crowd engagement or entrainment with the event (Collins, 2004, 2014), whether people watch games (or not) by themselves or with other groups of various sizes, any past or general interest in football (including the national team), and the degree of any opposition to or resistance with regard to the goal of being a good tournament host. The combination of all of these factors may be required to create a genuinely positive, engaged and seemingly spontaneous widespread emotional interest and investment in a national team’s performance. Moreover, where a national team performs well and is also the host of an excellent tournament, then conditions appear to be even stronger for widespread and intense positive collective emotion to be experienced (e.g., co-host South Korea in 2002; Horne and Manzenreiter, 2004; Germany in 2006; Sullivan, 2009). Research on national team fans, however, suggests that the emotions surrounding success and failure in a World Cup tournament are fleeting. Jones et al. (2012) found that when compared to fans of the failed English team, emotional responses reported by Spanish supporters to World Cup success were “more enduring and persist[ed] over 4 days after the event” (p. 168). They also note that further studies should include field-based research and “explore identity in non-sporting contexts” (p. 168).

### Specific Relations Between Embodied Individual, Group-Based and Collective Emotion Processes

Although self-report studies are common in the social psychological literature on group-based and collective emotions, in-depth qualitative interviews can provide valuable insights especially when embodied features and aspects are combined with participant observations of ritual and other features of people’s actions (Doyle McCarthy, 1994). Consistent with research demonstrating that using words such as “pride” can alter people’s posture (Oosterwijk et al., 2009), participants in interviews about positive group events and outcomes often reproduce experiential and expressive features of those emotions. Research on expressions of pride and personal triumph (Tracy and Robins, 2004) has studied them in individual rather than collective terms and contexts (Sullivan, 2007) despite collective pride being embodied and expressed by individuals in the largely the same ways (i.e., arms raised in triumph, expansive gestures, impulses and actions to share and celebrate with other group members). While group-based pride may be experienced alone or with others in prosocial I-mode, we-mode collective pride is likely to be reported as more intense than feelings about personal achievements; that is, people often report feeling personally “boosted” by experiencing a group-related victory or triumph particularly when with others and it is often the historical significance of an event “for us” that contributes to the intensity of collective emotion (Sullivan, 2014a,b). The research reported below therefore explores how feelings of group pride are embodied and reproduced in sharing-type conversations that draw upon personal and collective narratives of social occasions (Sullivan, 2012). Although participants in qualitative research such as Abell et al.’s. (2007) did not enact forms of national pride that have transgressive elements like boasting or arrogance, nevertheless the emotions displayed and verbally expressed in interviews can be intense and this is inferable also from claims and statements that indicate little empathy for opposing groups, reports of a sudden increase in status with regard to other groups or suggestions of defiance, hubris or arrogance (e.g., “this proves our superiority”; Holbrook et al., 2014). Connections with previous experiences of positive collective emotion or group solidarity are also likely to be recalled pointing to the potential relevance of collective memory research. Interviews represent an underutilized opportunity to explore the way in which they event is re-experienced when shared with an interested, empathic, but—in this case—outgroup researcher. Moreover, when combined with video analysis they allow interpreters to reach agreement about the meaning of embodied features of the communication and sharing of emotions (e.g., gestures, verbalizations, etc. Mascolo, 2009). Important insights about collective emotions can also be discovered by looking for patterns in individual emotional styles and group affective practices (Wetherell et al., 2015) and examining how features of geographical and social space afford complex, ambivalent and unexpected emotions. By including descriptions of the collective emotions manifest by large crowds and their subsequent impact on individuals, the case study will provide vital details about an issue that Tuomela (2013, p. 23) mentions: “bodiless group agents do not blush when ashamed, although their members may take part in collective guilt or pride and in similar shared emotions in the we-mode.” Thus while groups “can never be the full-blown agents (or persons) in the flesh-and-blood sense” it is possible to investigate empirically how they “share some similar functional features with intentional human agents” (Tuomela, 2013, p. 23).

### Study Aims

The aims of this study were to critically examine theoretical explanations of the emotional experiences of individuals and the collective emotions, behavior and actions of groups, specifically citizens of South Africa, in relation to ongoing national events in the country attended to by the majority of group-members. The chosen international event of the 2010 FIFA Football World Cup reflects unique features of the national group and its history of relations to other groups but also can be used to highlight features shared by many other events that generate national-level collective emotions. This complements research on crowds (Drury and Reicher, 2005), social movements (Simon and Klandermans, 2001), “banal” (Billig, 1995) and “ecstatic” (Skey, 2006) nationalism and patriotism, and the impact and legacy of mega-sport events. A further aim was to identify features of the event such as crowd interactions and reactions discussed by participants as emotional “peaks” and explore connections between participants’ emotions embodied in their reactions, gestures and actions as they recounted their experiences in interviews. A particular interest was to explore collective emotion themes at different periods during the World Cup: that is, before, during and anticipating the end of the tournament.

## Materials and Methods

### Study Context and Participants

**FIGURE 1:**
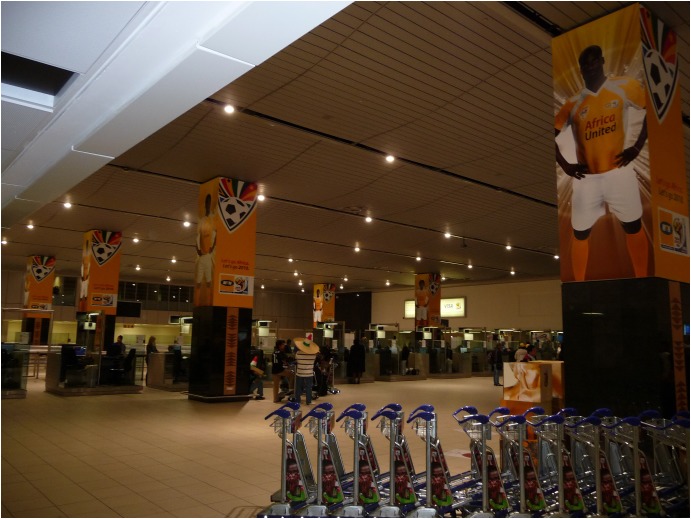
Africa United “proud” displays in airport arrival space.

**FIGURE 2:**
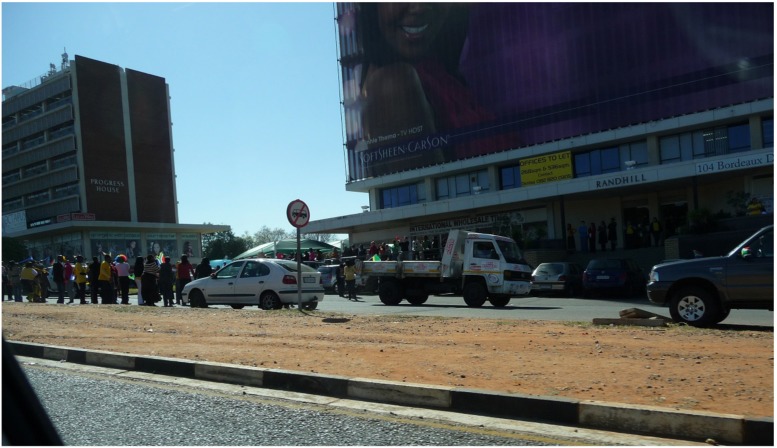
Initial groups of large enthusiastic supporters in Johannesburg lining the road before the South African football team parade.

When South Africa won the right to host the 2010 FIFA World Cup in 2004, it was described as a victory for the African Continent with the potential to create an “African renaissance” (Desai and Vahed, 2010). The tournament was keenly anticipated as an opportunity to promote international interests and investment in South Africa despite concerns about the bidding process, the cost of hosting the tournament, and persistent stories that the event might be moved to another country due to preparation delays (Ndlovu-Gatsheni, 2011). Johannesburg was an important focus of the event and this study because it was the location for the FIFA World Cup Kick-Off Celebration Concert on June 10, 2010 as well as several group games and the opening and closing games of the 2010 World Cup. Several public viewing sites were established in the north of the city, in the affluent area of Sandton, in the Johannesburg downtown marketplace and in Soweto. The research was undertaken in these and other locations chosen to maximize observations of the impact of the tournament (along with brief visits to Durban and Nelspruit). Data collection began in the week before the World Cup 2010 (see **Figures 1**, **2**) and finished the day after the World Cup final. Participants were 10 South Africa residents during the World Cup (see **Table 1**; one interviewee’s demographic information and interview content are excluded due to equipment failure).

**Table 1:** Qualitative interview participant information.

Pseudonyms	M1	M2	F1	M3	M4	F2	M5	M6	F3
Age	51	45	28	26	24	29	25	31	48
Gender	Male	Male	Female	Male	Male	Female	Male	Male	Female
Nationality	South Africa	South Africa	South Africa	Lesotho	South Africa	South Africa	South Africa	South Africa	South Africa
Occupation	Taxi driver	Unemployed	Student	Student	Student	Administrator	Student	Student	Assistant in Bank
Cultural/Ethnic Identity	Zulu	Zulu	Zulu	Black	Mbeti	Tamil-speaking Indian	Tsutu	Indian	White
Previous interest football	Yes	Yes	No	Yes	No	No	Yes	Yes	No


### Method and Analysis

**FIGURE 3:**
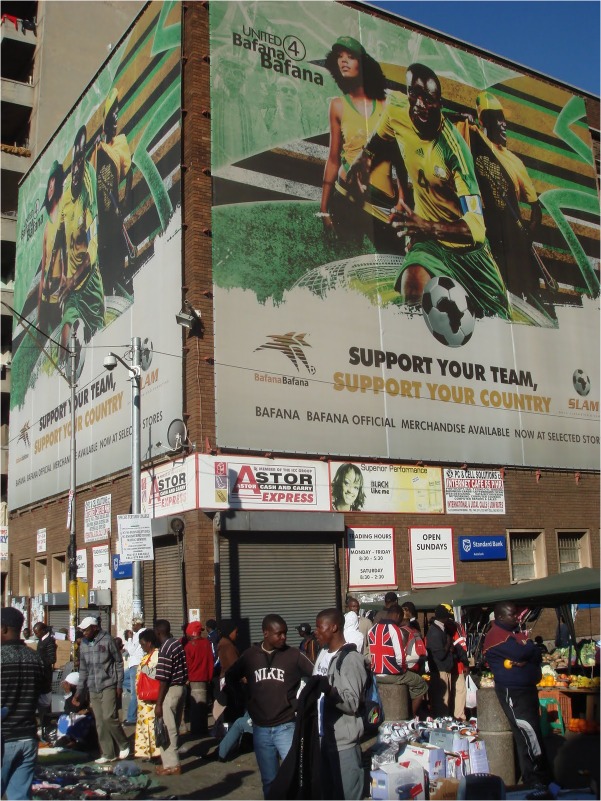
Example of widespread small narratives indicating the normativity of support for the national team and national interests.

**FIGURE 4:**
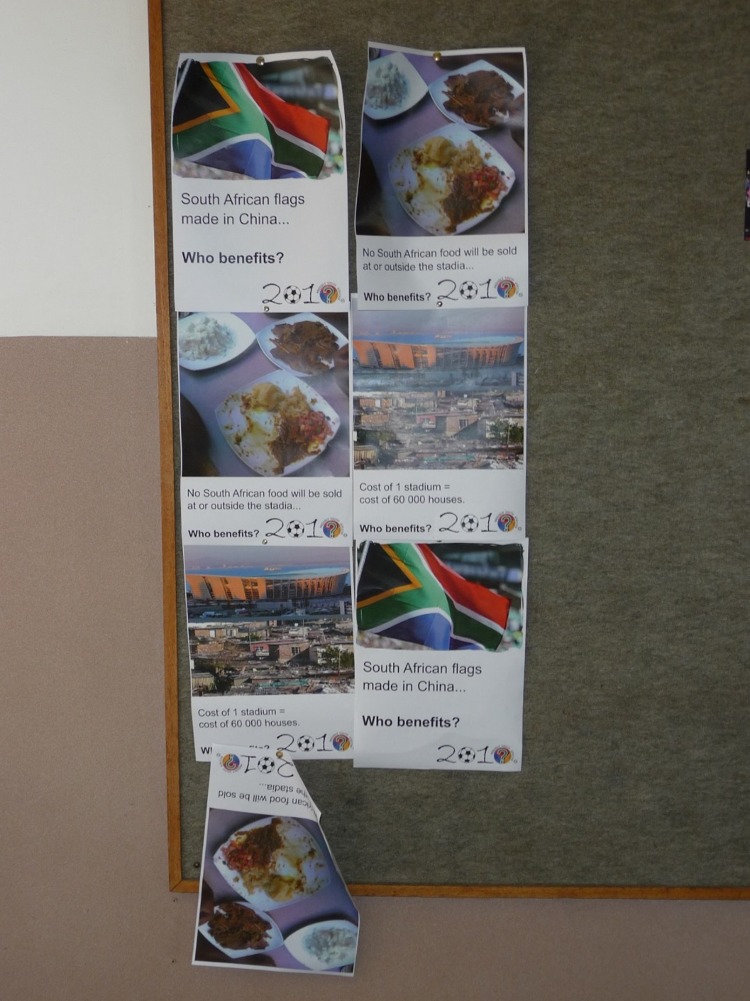
“Who benefits” posters critical of the World Cup displayed on the University of the Witswatersrand Campus.

**FIGURE 5:**
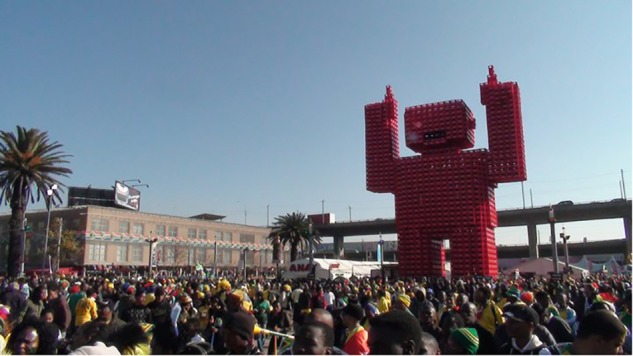
Crowd and sculpture at Fan Fest in downtown Johannesburg.

**FIGURE 6:**
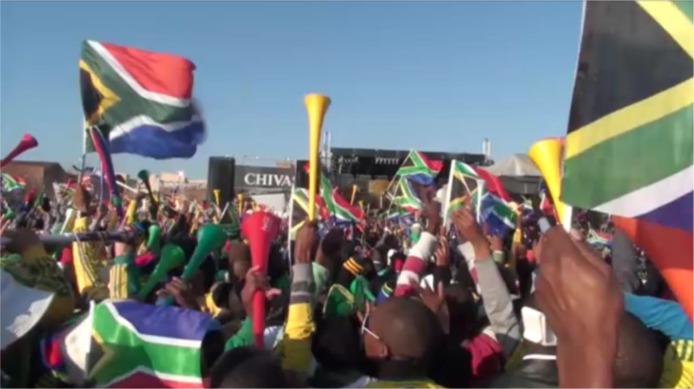
Photo from author’s video of people in Mary Fitzgerald Square singing the South African anthem before the opening game.

The case study was approved by Monash University Ethics Committee. Participants were provided with printed or verbal participant information and their full and informed verbal consent was recorded. The study used a critical realist framework rather than a social constructionist or discourse analysis approach because while it was important to examine instances of emotion talk in action—including how they were used to work up notions of unity or mobilize previous instances of everyday national attachments—and knowledge claims about the realization of emotions in large groups. A further aim was to triangulate multiple data sources to make robust claims about how and where collective emotions occurred and their dynamic features over time. This resembles the case study method employed by Stott et al. (2017) to understand the 2011 riots because the sequence of events is used to frame the thematic analysis of pre-, during- and anticipated post-World Cup events. The multi-method case study combined the qualitative methods of participant observation ethnography (Hammersley and Atkinson, 1995; Sullivan, 2012) with news and social media and in-depth qualitative interviews typically lasting between 30 and 60 minutes. Participants were recruited through posters displayed around the Witwatersrand University campus and in the field during naturally occurring conversations in public settings. Publicly posted Youtube videos were examined and a Guardian newspaper video (GV) of edited interviews with South Africans (4 women, 7 men; posted on the now defunct https://www.theguardian.com/football/worldcup2010 webpage) was transcribed and analyzed because it included rich first-person accounts that complemented the study interview strategy of purposive sampling in order to maximize participant variation in terms of age, gender, ethnicity, class, interest in football and political opinions. The principal investigator recorded high definition video of public spaces and interviews (with participant permission) and photos of representations of South Africa and the World Cup 2010 in Johannesburg (see **Figures 1**–**6**, **8**–**10**). Field notes were written down for subsequent analysis after visits to public sites such as malls, markets, and Fan Fests. Ethnographic observations initially focused on downtown Johannesburg and Sandton before other suburbs and places were explored in order to maximize variation in South African citizens’ practices of engaging emotionally with and resisting the World Cup. Reflexivity was central not only because the collective emotions, celebrations and behavior of people were being explored from the “outsider” perspective of a white Antipodean male—which made cultural comparisons possible and also freed some participants to be critical of their government and fellow citizens—but also through critical engagement with the theoretical literature in order to identify and empirically examine any taken-for-granted assumptions about what collective emotions are as well as how and why they occur. Critical reflection on the interdisciplinary collective emotion literature, nation-related affects and their relevant spaces informed the observations undertaken and the in-depth semi-structured interviews focusing on the experiences, observations and practices of participants. In a further innovation, five of the interviews were videorecorded so that previously unexamined features of the embodied and expressive could be explored. The data were analyzed by considering the sequence of events and exploring the observations and interview accounts during the event while also observing and talking about key emotional experiences during preparations and in the days leading up to the opening ceremony. Thematic analysis (Braun and Clarke, 2013) of the nine verbatim interview transcripts and the GV transcript provided the framework for coding, disconfirming examples were actively sought within themes and criteria for good quality qualitative research were followed.

## Results and Discussion

Many participants confirmed not only that they witnessed a range of strong, widely shared group emotions during the World Cup, but also immediately before the start of the World Cup. The following sections, therefore, analyze accounts of collective pride and related collective and group-based emotions reflecting national interests and identity positions in two key periods: before the World Cup opening and during the event. The latter included people anticipating the end of the tournament, their accounts of the long-term impact and legacy of the World Cup and their emotion-laden and evoking narratives about South Africa’s future. The aim was to represent not only the affects that were prominent in both periods, but also to explore specific examples and reports of embodied reactions derived from collective participation which include group-based examples of hubris and possible contempt (Sullivan, 2017).

### Collective Emotions and National Pride Prior to the World Cup

In many previous investigations of World Cup host nations, there have been few reports of collective emotions before the event. Rather, there are often criticisms and concerns about issues such as ticket prices, infrastructure costs and event security as well as increasing local prices, traffic jams and overcrowding. In contrast, South African participants reported levels of interest and excitement about the imminent beginning of the event that could barely be contained. Although this arguably reflected top-down narratives to support the event in accordance with advancing national interests, a more immediate sense of bottom–up spontaneous and spreading enthusiasm was evident This was demonstrated on the day that the author arrived in Johannesburg and traveled by car toward the center of the city. During the trip, large excited crowds had already gathered on the side and middle of the road for a parade to welcome the South African national team, “Bafana Bafana” (see **Figure 1**). People danced, cheered and waved at anyone driving along the street; this was before the parade had started. Accounts of joyous celebrations and dancing and an infectious mood were also mentioned spontaneously by several participants, although remarkably none experienced the parade directly:

M3: Yah I heard about that yah I was there in spirit but yeah not physically, it was [inaudible] or was everywhere and no one could really resist it, it was tempting so everywhere you feel like blowing the vuvuzela you know, flying the flag high, the South African flag yah that was fantastic

For another participant this was mentioned as one of several times when she felt national pride: “Um firstly when our team paraded down in Sandton, I wasn’t there but we all watched on television” (F3). Participant M6 confirmed the turnout for the parade “was something like when Mandela was released from prison” and “I’ve never seen so many people take to the street and of different races just like together, enjoying it.” She spontaneously contrasted this with other accounts of race-based support for national rugby and cricket teams. What is striking here is how participants felt a shared excitement that was widespread: the parade “made everyone realize like wow, everyone’s backing them up, everyone’s just waiting to party [laughs] and celebrate and enjoy the tournament” (M6).

The suburb of Sandton was not easily accessible to several participants for a variety of reasons and that they had missed a significant occasion: “I really wanted to go and I think that was the first day that I actually like really got into this World Cup thing” (F1). While it is not unusual for parades to generate enthusiasm, on this occasion the anticipation of the event appears to have been the catalyst for large group celebrations and expressions of excitement and enthusiasm. The ritual of the parade provided facilitated celebrations among crowds who wanted to assemble to show their strong and widespread support for the team and express excitement that the widely anticipated event had almost arrived. This is different to the type of slow build-up that has previously been experienced by host nations of megasporting events such as the Olympics, which tend to start with a mixture of anxiety, concern and skepticism that gives way to enthusiasm. For those participants who could not go to Sandton but listened on the radio, they were either there “in spirit” (M3) or, in one case, created their own parade:

M4: on our side like in Jo’burg downtown ahwah I-ah it’s different from Sandton, downtown like a lot of them, like they are not South Africans and the South Africans who are here, mostly are students and some are just ahh normally, normal workers like yeah but we had our own parade ahhuh even though Bafana was there, we had our own parade in Bafana shirt and like the feeling yah

This illustrates the potential to create a local variation on a ritual which is not possible to participate in for reasons which are about limited resources and exclusion, but also show the desire of people in the Newtown area to create their own version of the event. Accordingly, the parade was not experienced as a denied opportunity for this community because they were still able to share in the exciting events organized around Sandton.

From the Guardian video, a white middle-aged women confirmed my experiences and others’ rich accounts of the Sandton parade when she recounted how she shared the experience with another close acquaintance after not initially intending to go:

GVF1:… we went there and I don’t think one can describe the atmosphere, the people were buzzing, but there were no barriers of colored-colors, color-black, white, we were South African and um to stand there and feel it, they were actually dancing in the streets and we waited quite long for the Bafana players but I can tell you [puts hand on heart] I had “goose flesh”

The example shows the combination of the “irresistibility” of the parade and the subsequent feeling that this was an inclusive celebration. The intensity of feeling is indicated by her smile while retelling the story and feeling goose bumps (i.e., piloerection) while in the crowd. Vuvuzelas contributed to the cacophony of noise but what was extraordinary about the parade for many South Africans was that this did not feel like a gathering of support for a national team that was divided along racial lines. Although this participant did not join in the dancing, she still experienced it as part of a kind of extraordinary carnival; a unique celebratory and inclusive solidary atmosphere.

### Other Factors Contributing to Pre-world Cup Celebrations

An American visitor who posted his video on Youtube (Levitt, 2010) exemplifies the strong impression to an outsider that the World Cup has already brought people together. Observing a large banner near the Bafana advancing team bus in Sandton “UNITED WE SHALL STAND,” he remarks: “You see that? That is for sure. This whole country is united for this.” The example also shows how large excited crowds can be taken, sometimes erroneously, as evidence of widespread emotion and enthusiasm across a whole country. Ubiquitous small narrative phrases and images evoking national unity, support and pride (e.g., see **Figures 1**, **3**, **9**, **10**) also framed occasions and spaces, indicating the top–down normative expectations of the South African government, corporate sponsors and FIFA of citizen support for the national team and pro-African sentiment during the tournament.

Further evidence of the ubiquity of group norms of national pride, support and unity comes from rare public examples of counternarratives and instances of personal resistance in public to the World Cup within the Witwatersrand campus (see **Figure 4**). Several participants indicated how the World Cup going ahead was a positive response to internal and international critics. With regard to internal critics of the World Cup and accepting some of these, M5 concentrated on problems with the claims made for the impact of the event on the lives of ordinary people such as opportunities for employment: “they’ve been mentioning a lot of things that I would say, not even the half of it has been accomplished.” Many participants found such points harder to articulate during the World Cup as we shall see below and the issue of internal criticism was raised also with regard to considerations of the impact and potential legacy of the event. Before turning to the emotions that we

M3: I felt so bad about those criticisms because I felt maybe there has been another sentiment to underrate to overlook Africa as a poor continent, the Africans as lacking capability to organize such a big event … that’s really generated mixed feelings to us as Africans because we were really expecting it, then that’s really obstructed our potential, obstructed our expectations, we thought maybe ahh FIFA will eventually drop South Africa as being the place where this tournament will be hosted

Such complex and mixed emotions are consistent with findings of ambivalent pride and shame co-existing in everyday life (Miller-Idriss and Rothenberg, 2012) but especially simultaneously occurring in-group pride and out-group directed anger (Becker et al., 2011). Some participants demonstrated this complexity once the tournament had began with their vivid evocations and imaginings of critics having to say that they were wrong; that is, to admit that South Africa could host the World Cup and welcome international visitors (see the final section on anticipating and discussing the long-term impact of the tournament).

In subsequent sections, indications of the limits of any sense of the World Cup being a joyful celebration across all regions and ethnic groups in South Africa will be challenged. It is important to note that while a parade is a good example of a ritual that can encourage people to show their support and enthusiasm, the Brazilian coach of the South African team remarked that the parade was like the type of celebration that would normally occur *after* winning a World Cup rather than before the team’s first game. In this regard, the team parade provided an opportunity for people to show their support for the team reinforced before the tournament by public campaigns showing happy, multiracial crowds and evoking a norm of united support for the team and for celebrations of South African collective identity (e.g., as represented in **Figure 6**). Most of the crowd waved South African flags or were dressed in the yellow and green colors of the South African national team, reflecting calls before the tournament for people to wear the national colors. For example, as noted above businesses encouraged people to see the team parade at lunchtime, but also as “Football Fridays” which F1, M4, M5 and F3 all described as occasions over several months when they were expected to wear national team colors at work. Several participants reported that this weekly practice reminded them of the approaching tournament and helped to generate a feeling that “it is finally here” when organized events took place and the arrival of football stars confirmed the start of the tournament was imminent. The mood amongst citizens of Johannesburg prior to the World Cup contrasts with news narratives and interactions with others at several other events directly experienced by the author, including the Sydney 2000 Olympics and the 2012 London Olympics in which there were no celebrations before both events that were in any way comparable to the celebrations during the Bafana parade on June 9th and observed by the author on the streets of Newtown in the hours before the televised FIFA “Kick-Off Celebration Concert” in Orlando Stadium, Soweto on June 10th. This event also created much enthusiasm as international music stars mixed with local musicians for a global audience and several participants reported beginning to engage emotionally with the World Cup at this point. Accordingly, the next section turns to experiences during the World Cup, including accounts from participants who attended public viewing spaces.

### Group-Based and Collective Emotions During the World Cup

Although several participants reported awareness of the parade and street celebrations in downtown Johannesburg, significant experiences for many occurred while watching the tournament with others. This varied from viewing games and key moments (e.g., FIFA concert) on television at home with friends and family to Fan Fests in Johannesburg such as those official organized in Newtown (Mary Fitzgerald Square in downtown Johannesburg, Sandton, and Soweto) with respective capacities of 22000, 20000, and 40000 people (Official Website of the City of Johannesburg, 2011). Nine further township Fan Fests operated under the name of “Township TV” in parks with viewing capacities in each “safe, secure environment with 24 h security” varying from 1000 to 6000 people (Official Website of the City of Johannesburg, 2011). Experiences of the author at several Fan Fests are reported below along with interviewee accounts of their experiences and evidence from online videos and news media.

### Opening Ceremony and Anthem as Sources of Pride and Unity

While there are no details available on the percentage of the population who watched the opening ceremony and first game of the World Cup (in contrast, for instance, to statistics available for Germany in 2006; Sullivan, 2009, 2014a), many participants noted that they watched this at home on television with friends and family or viewed it at one of the new FIFA Fan Fests. For participant F1, it is significant that the Bafana parade and then the experience of singing the South African anthem at a Fan Fest overcame her ambivalence and concerns about the event:

F1: I feel like kind of souuuuurr I mean even though I have my reservations, it’s still here [inaudible] like at the opening it was still a good time

The novelty of the public viewing Fan Fest in South Africa contributed toward a new collective emotion experience for many participants:

M3: Yes this was a new thing in a way and for me it was such an amazing experience as well, this was a phenomenon because I was there actually, the opening of the games, I was at the Fan Fest, it was my first time you know I was “ehh” this is truly truly an African World Cup, we were there together mixing with other people from Mexico from different corners of this planet and I was really over the moon and for me having that experience, people being together, cheering together, watching the big screen

For one participant, this was a further occasion in which she felt national pride which she re-experienced in the interview:

F3: the opening ceremony was phenomenal um people all wearing yellow t-shirts, you just thought oh I’m a South African you know normally you’re kind of like not really you don’t really show where you’re from because of our problems, whereas, the Fan Fests were, you could spot the South Africans and felt proud to be South African

Remarks about “the way we handled the World Cup” (F3) and generally “knowing we could do it” were reflected in several comments which are all captured by one participant’s reworking of a commonly used advertising phrase during the tournament: “We were waiting for it 3 months back, we know that slogan ‘Feel it, it is here’; we were feeling it, it has arrived now” (GVM2).

With regard to emotional peak experiences, in the Guardian video a young black woman noted,

GVF2: The best part for me was the national anthem, I went to the fan park just to sing the anthem with everyone and it was, wow!

In my interviews, a participant indicated that the crowd signing the national anthem at the opening ceremony was a particular source of national pride (see **Figure 6**):

M1: Ahhh I did experience national pride when our people were singing the national anthem you could see that people were singing from their hearts, from the bottom of their hearts so it has just brought us together, black and white like you’ve never seen before
I: Really, that’s what it feels like to you?
M1: Yes in terms of unity that you’ve never seen before

The connection to unity is important here, and specifically reports of a “felt unity.” There was clear evidence that seeing and hearing others signing the national anthem and supporting the team *en masse* in a manner not previously experienced was potentially transformational for participants who were used to racial divisions. The anthem and, as will be discussed below, the South African team’s first goal were consistently mentioned as points of intense, shared emotion. No features of the content of the opening ceremony created sustained emotions, although images of Nelson Mandela and Desmond Tutu were cheered and there was muted discontent when the former President and Vice-President of South Africa F. W. de Klerk appeared on the big screen at the Fan Fest I attended.

While social identity theorists like Kuppens and Yzerbyt (2012) might argue that the anthem not only demonstrated the salience of a South African identity but also celebrated a new passion for this identity, a we-mode analysis emphasizes that people were afforded a rare opportunity to show their pride through acting *as a group.* For some people this was their main goal and although this could be interpreted as a desire to feel a group-based positive emotion or have a pro-group I-mode experience (as I did by being impressed by the singing of the anthem but without otherwise participating in it), the bulk of the evidence suggests that it was a “full we-mode” collective emotional renewal of commitment to group goals and ethos. In this respect the corresponding feelings of unity might resemble the collective empowerment social and political activists feel when they chant together and assemble in public spaces. However, these feelings generated collectively by participating in a successful collective action contrast with the experience of success provided by the team within the contingencies and uncertainties of a game of international football.

### The Euphoria of South Africa’s Opening Goal

An emotional highpoint in the opening game was South Africa’s first goal against Mexico in the 54th minute. My experience at the Newtown Fan Fest of wild celebrations was reported also by several participants and is evident in online videos. When the first goal was scored in the opening game, there were reports of widespread euphoria:

GVM2: It was a magnificent goal, people were just going mad, like I was hugging people, screaming, people were playing vuvuzelas, jumping, were tackling each other with (1 sec.) I lost my voice that day, like I couldn’t speak for the next week or so because of how much I screamed just at that one goal

**FIGURE 7:**
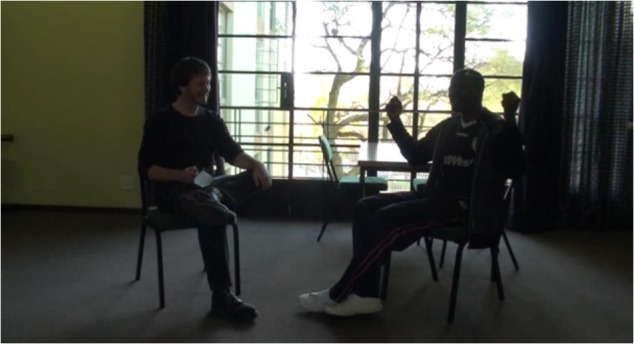
Interview participant expresses and re-experiences pride when talking about the opening game of the 2010 World Cup.

Participant M3 described similar feelings and also re-experienced the elation in the interview (see **Figure 7**):

M3: Wowww [laughter] I remember I was like, I couldn’t really hold-I kept screaming um cheering with friends you know people [who] were around me we were so excited, euphoric mood, you couldn’t really resist that, we were celebrating, jumping you know ahh that was a great goal and you know I was so happy

Displays of individual celebratory behavior and elation when group interests are secured or a collective aim is (temporarily) achieved resemble those of individual pride (e.g., arms raised outward) and these are reproduced in conversations about the events concerned (e.g., they could be said to be internalized or appropriated). However, when experienced in groups the celebrations increased the intensity of individual emotions and the noise of the crowd celebrating jointly created a mutual amplified collective emotion (i.e., a full we-mode celebration). As indicated by the participants and as experienced by the author, there is something special about overcoming usual physical barriers between strangers in a crowd to celebrate with others by hugging, hi-fiving, and interacting with others.

**FIGURE 8:**
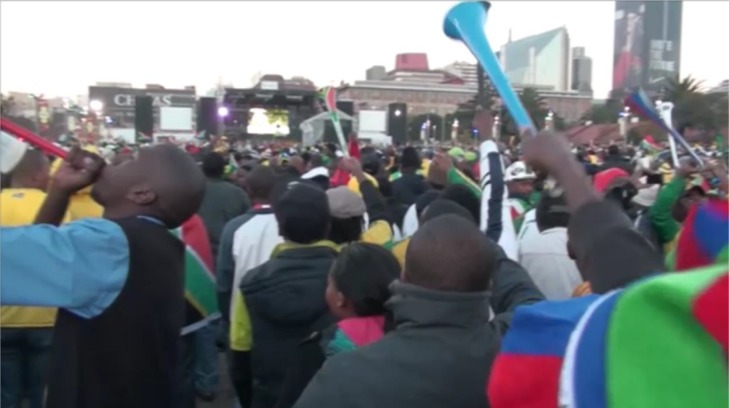
Crowds continuing to dance, sing and play vuvuzelas after South Africa’s first goal in a World Cup.

Although this might be described in terms of contagion, a better explanation could focus on a combination of the action tendency to share such moments in highly physical celebrations, the impressive sight of seeing large numbers of people celebrating intensely, as well as the effect of contributing to the emotions of a noise of a celebrating crowd. The importance of shared goals, a history of interest in the event and relevant embodied cultural knowledge and practices is demonstrated also by the author’s happiness for the South African viewers and inability to emotionally match and physically join in the intensity of the crowd’s celebrations. I was also surprised to see many people in the Newtown Fan Fest turning to celebrate with each other after the goal and that continued to dance and sing together far beyond what usually counts as a celebration for people in public viewing sites in the United Kingdom even with an important game. It was unclear whether these may have been groups were part of existing established networks (e.g., of friends, colleagues) or were spontaneously formed. The positive mood for those supporters at the Fan Fest who maintained some focus on the ongoing game was only punctuated by Mexico scoring the equalizer. This provoked whistles of disappointment and an audible groan, but still did not stop many of the dance-based celebrations (see **Figure 8**).

### Further Feelings of Unity and Togetherness

The opening ceremony of the World Cup immediately proceeded the first game. Many participants felt that it was important to watch the game at a fan zone or with other friends or family members. A young black man in the Guardian video described how the “first game was fantastic, most of the people were chanting like brothers” and noted “since I was born I’ve never seen people doing chanting like that” (GVM1). It is highly relevant to this account that one participant highlighted the shared goal or desire of the crowds during the opening game which overcame racial divisions:

M5: We all wish that Bafana Bafana could win, you know, we all wanted to win, but at that thing, you know I think, I feel as that we become friends because we have something in common so that thing made all of us friends, nobody hates anyone, irrespective of the color of his skin

Unity was also experienced by some participants as a direct result of being together in large multi-ethnic South African crowds. Several participants felt that the ethnic divisions which persisted in the country despite the end of apartheid were temporarily overturned in fan fest crowds along with differences between locals and foreigners:

M3: I mean sort of unity ummm seeing ah whites, I mean black people, Indians and foreign people together that was something remarkable you know because we are having this, I think it’s called racist tendencies um I think yeah amongst our people is still a problem, but seeing people joining hands together not looking at the color of the skin um enjoying soccer, sharing together, giving war- a warm handshake that was a different experience

Being with “friends” sharing a common goal was therefore a powerful experience for many participants, suggesting a unity that they wished could be experienced more in everyday life.

In some cases, the impact of watching with others was evident in the “conversion” of people who were not supporters of football to the “cause” of the South African team but also potentially to a more inclusive South Africa. On the first issue, some South Africans who normally did not watch football appeared to have been influenced by the emotion surrounding the first game such as one middle-aged white woman who remarked: “My first soccer game I’ve ever watched was the game with Bafana and Mexico and now I’m a fan, a big fan”. An implication was that this process happened suddenly through wanting the team to win rather than, identifying closely with them as players:

F3: There’s suddenly all these new emotions [are coming] [gestures of both hands toward her head] and you’re standing there thinking [hands up in the air] support for a team-you don’t even know who they are, but you’re supporting and you’re feeling this love in your heart for them and you just wish that they had gone further, you feel sad for them and that they didn’t, but delighted that we actually had a showing, and proud of them, very proud I think that’s the main thing, the proudness of South Africans suddenly and I think most people, as I said, feel the same as I do

**FIGURE 9:**
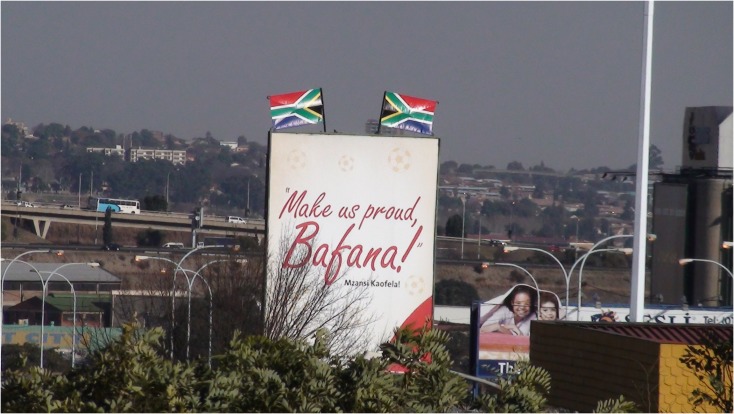
Advertising reflecting expectations of Bafana Bafana as a source of national pride.

It is worth noting here that this rich account is somewhat consistent with accounts of banal and “ecstatic” nationalism in the sense that ordinary notions of South African national interests in everyday life (including collective memories and cultural practices) not only create dispositions that might be “mobilized” or re-constructed as appropriate to show on particular occasions, but also are evident as practices that are saturated with affect and newly experienced desires in sometimes spontaneous and surprising ways. The practices through which national identities are reinforced and expectations about behavior are represented (e.g., see **Figures 5**, **6**, **8**, **9**) can then create a sense of pressure to conform one’s pro-group behavior and comply with the expectations and wishes of others. But they also suggest that the ways in which national identities are constructed can be manipulated to generate support very quickly for a team representing the nation to succeed and, indeed, creates or realizes a previously not consciously known desire for the whole nation to experience a widespread celebration (i.e., the fantasy of what it would feel like if “we” or another African nation won the World Cup).

Once the World Cup was underway, several participants reported increased feelings of pride and experienced a heightened sense of national identity. In one case, this was despite not participating in some of the practices that would usually enact support and predispose participants to become emotionally entrained in national team games:

F1: Ah well [laughs] yeah I felt that I’m South African more, and I know
I: Really, South African more?
F1: I don’t know how to explain it though, like I didn’t have a flag on my car and stuff but certainly I did support the South African team and there’s the whole getting together as a nation to, you know, root behind our team

Consistent with an approach to qualitative research in which dissenting accounts are also analyzed, a male middle-aged interviewee argued that colored (a term commonly used in South Africa to indicate a person of mixed European and African or Asian ancestry) people like himself are second class citizens and did not benefit from the World Cup: “All the jobs and the opportunities goes to the black people, you know, and we still come second, so what did we achieve in the whole thing [shrugs] nothing” (GVM4).

### Normative Support and Limits to the “Spread” of Positive Collective Emotions

Extending the notion that group-based positive emotions encourage sharing and, in some cases, celebrating with other people who might otherwise be strangers or out-group members, collective positive emotions also generate feelings of group solidarity, encourage people to celebrate in public spaces that have temporarily been taken over and lead to revision of group narratives. But people in a positive mood may overestimate the level of interest in a group in part because the action tendency to share and celebrate with others is intrinsic to euphoric and intensely positive emotions. At such points, it is possible for expectations of widespread similar emotions to inform people’s expectations about the experiences of others. For example, one participant implied that feelings of collective euphoria, pride and unity were shared throughout South Africa only to find that these were not occurring in his home province. This led him to state: “World Cup thing has benefitted Johannesburg more than any other provinces, I was so surprised seeing like my family not even watching” (M5). This appeared not simply to be a failure of contagion-like spread of emotion, rather his home province was like another country: “it’s more like you are now not in South Africa because there in Johannesburg, you are in South Africa, you can feel the vuvuzela, you can feel the spirit like when they’re going to play, I can see people are wearing gears all. just all, so you know, but there it’s just quiet” (M5).

Despite the euphoria of a South African goal in the opening game of the tournament with Mexico, Bafana Bafana’s subsequent loss to Uruguay and departure from the tournament after an anticlimactic win over the fragmenting French team heralded the end of interest in the tournament for several participants and potentially a large percentage of the population. In relation to the second game, one participant noted that the team had let him down:

M2: because I didn’t realize they can lose a game like that, so my heart was broken down, I was hoping that maybe they can nearly go to the quarter final, semi-final, hmm hmm out so early

While this exit of the host nation from the tournament reduced the possibility of the kind of euphoria that accompanied the national transformation of the German host nation during the 2006 World Cup or indeed, was integral to the unifying impact of the South African Rugby team’s World Cup tournament win in 1995 (Møller, 2014), several participants spoke of some ambivalence about continuing to display support and, specifically, to wear the South African team shirt. One spoke of a visit to Durban where people weren’t still wearing shirts:

F1: when Bafana played their last game and I was wearing my t-shirt and my cousin said “oh are you still wearing the loser’s t-shirt” and I’m like I’m still like “whatever you’re still South African, they’re still our team” so they lost but maybe they’ll win this time that’s kinda-like I wanted to take it off, but yeah I carried on wearing it

In a similar vein, a collective instance of mixed emotions appeared to occur among people attending public viewing for Bafana Bafana’s game final group game against France experienced a mixed emotional atmosphere. In a Fan Fest I attended, men were mostly subdued about the slim possibility that South Africa might score enough goals to progress, whereas many women celebrated the team’s victory seemingly unaware of the implications for the team in the tournament. On the Guardian video, a young black man yelled a succinct summary after this last game for Bafana Bafana: “finished, it’s finished, gone, finished.”

Several participants were able to switch support to other African teams as a possible source of a pan-African pride: “The games themselves were out of this world, I was just disappointed as an African that none of our teams went through to the semis” (M1). The loss of possibilities to celebrate an emerging successful African identity should not detract from the emphasis some participants continued to place on the value of hosting an internationally respected tournament:

GVM2: I was sad but I feel that Bafana Bafana is not the one who host the 2010 World Cup, it’s we South Africans who host the World Cup

As the next section highlights, the affective impact of the World Cup for many South Africans was the sense that hosting the tournament had improved the status of their nation within an unquestioned ideology of leading and developing nations. This included the recognition of some participants that they themselves doubted whether the tournament would be a success: “I was skeptical because I never thought we have the infrastructure, transport, communications, everything, it’s such a big event [raises shoulders and opens hands outward] I never thought we could host so many people at one stage” (GVM4). It is also important to examine how people respond emotionally to the end of the carnival-like feature of mega-sport events and the resulting loss of the international spotlight.

### Affective Features of the World Cup’s Short- and Anticipated Longer-Term Impact

When people reflect on their memories as citizens of the host nation of a mega-sporting event, the most prominent stories are about emotional high-points and a sense of sadness about life without the World Cup’s month-long celebration of football and the drama of being the focus of the world’s media. People often orient toward the complex and mixed “impact” or “legacy” considerations that are emphasized in bid documents but which are not only difficult to investigate when the focus is on the “intangible” level of individual emotion—because these may be highly idiosyncratic and are often presented as dissipating rapidly—but also complicated when attempting to capture and chart the short and longer-term consequences of collective emotions at the macro-level of the nation’s newly forming memories. A generation-specific example of differences in experiences of national history and a potentially new shared memory is the following:

GVF2: I’m a 1982 child, so I wasn’t part of the Apartheid era which obviously made headlines, so for me to be part of the World Cup, which was a positive side of South Africa, it was a time for me to really stand up and be proud, it’s a part of my life that I’ll never forget, never ever. I’m sure [laughing, gestures to others] everyone can say the same thing.

Collective memories that may be evoked again in the future, shared and potentially re-experienced in those moments are important long-term features of the impact of a mega-sport event that may be used to mobilize support for other nation-building projects (e.g., a bid to host a summer Olympic games). GVM4 noted, for instance: “I think because the World Cup is such a big event, I think that this, for me, it makes me feel like we can achieve anything that we put this much focus on, this much money into.”

In the short-term, several participants reported anticipating sadness that the tournament would end and everyday life would return along with a possible return of problems such as xenophobia toward foreigners—“people are promising as soon as the World Cup finished we are going to beat and burn alive all the foreigners” GVM5—and new stadia become “white elephants” (GVM3). There were clear limits for some with regard to enduring social cohesion:

F1: it’s been a very nice boost, it’s been a very nice, but I’m still skeptical, we’ll see afterward, there’s hope, I think there’s hope for more unity. I don’t know how we’re going to keep it up though [shrugs]

This summary at least appears to be more positive than the doubts that some participants felt even after they had just left the Fan Fest such as future-focused concerns about how inequality in the country could be reduced (M5). Others worried about crime levels returning to pre-World Cup levels and whether it will be “back to square one” (M1) even if there were more overseas visitors than before. Nevertheless, there was some satisfaction that the tournament had overturned international expectations: “Everybody expected us to fail and I think we surprised [nods emphatically] the world” (GVF1). Accordingly, the collective pride that persisted after the tournament included the feeling that the tournament had changed international perspectives: “We’ve worked hard for this, we’ve worked hard for a different image to the outside world so please let not-let’s not ruin this” (GVF1).

**FIGURE 10:**
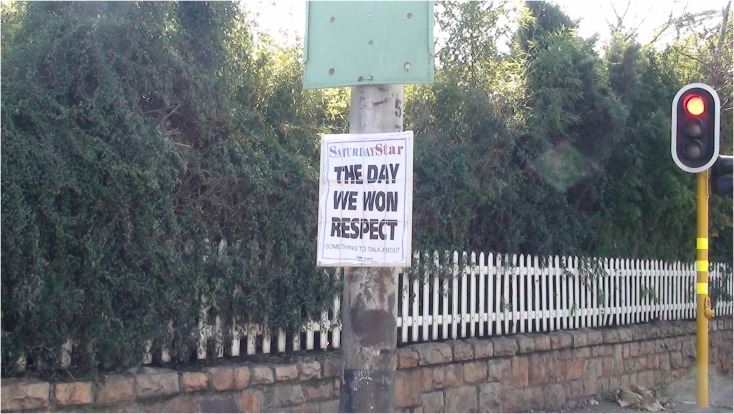
Newspaper headline the day after the opening game of the 2010 World Cup.

Focusing on the fact that the tournament had been delivered without obvious major problems also encouraged individuals to demonstrate some features of hubristic or arrogant group-based pride. For instance, one participant oriented toward the implied “disrespect” that had been addressed during the first game (see **Figure 10**) and which contributed to a sense of defiant pride that South Africa had proved critics wrong. She noted that of national pride,

F1: I think I’m still experiencing it now, that we were able to host this World Cup successfully, I mean the British newspapers, whatever, they were very anti in their support compared-I don’t know because CNN and American, if you compared the other one, BBC and CNN… were more pro-World Cup, pro-Africa, pro-Vuvuzela and the other side were more anti, so in a way it’s like [opens up palms outward and raises upward while smiling] hey, in your face, we’ve done it!

In this case, F1 appears to have been empowered by the tournament to assert a newly found confidence in South Africa and its improved international status. While very different from the transformation that the 2006 World Cup had on subsequent expressions of collective German national pride (i.e., from previous levels of shame and concerns about the risks of nationalism), this was further evidence of an imagined shift in South Africa’s image. In response to my question about whether the World Cup has changed anything about South Africa, participant F1 shifted from appearing to minimize the impact to an almost hubristic sense of South Africa’s transformed status:

F1: Well, no (1 sec.) in terms of the big things, in terms of international exposure, other countries are more open to South Africa, I don’t-I really- maybe we’re even moving away from being a third world country, yeah I feel like South Africa’s kind of the leader now of, of ahhm Africa

This example of an individual expressing a kind of empowerment based on their group’s achievements and the possibility that it could be seen as trangressive in imagining South Africa’s new status among African and international nations, it is important to note that such sentiments need to be widely shared in narratives about the nation and become accepted parts of the collective ethos in order to have the potential to create a genuinely collective form of hubristic pride (e.g., that might lead to enjoyment of this newfound superiority over and potential contempt toward other “non-leading” African nations;Sullivan, 2017).

## Concluding Points

The case study highlights the importance of studying mega-sport events as complex affective occasions in which feelings of widely shared euphoria, collective pride and a unified national identity are possible but not inevitable. The research showed that prior practical activities such as wearing national team shirts combined with narratives presented in multiple formats, (including everyday conversations) to provide the background to realizations of collective emotions in two main public spaces: the streets of Johannesburg and Fan Fests. This extended previous theoretical speculation by Kelly and Barsade (2001) focused on small groups to highlight the interaction of affective and non-affective factors responsible for collective emotions at the national group level. Orienting also toward international criticisms that the World Cup might not take place, many citizens of Johannesburg celebrated the start of the event in collectively manifest enthusiasm and shows of support that are usually not seen at World Cup or Olympic events. Ritual features of the event that were recalled as emotional peak experiences included witnessing and participating in singing the national anthem, the parade in Sandton and the television viewing of games (i.e., rituals of mega-sport event broadcasting; Sullivan, 2014b) all of which afforded collective emotions that mobilized previously constructed affective dispositions (e.g., toward the nation and which created also a sense that a transformation of South Africa’s status would be desirable and that it would be good for the South African team and tournament to impress the world). The experience of public viewing was transformational for many participants although the intensity of the emotions and change in collective identity was not comparable to Germany’s in 2006. Personal accounts of the “macro” national and international impact of were expressed in terms that showed how a national event might encourage feelings of hubristic or arrogant pride, that might even underpin contempt for other groups (e.g., other “less respected” nations in Africa).

In this analysis, the need to discuss levels of collective emotion was not so evident in the micro and meso-level focus reminiscent of Drury and Reicher’s (2005) research on collective empowerment. The transformational nature of experiences of mega-sport events are quite different to engaging in politically motivated forms of collective action. Following a team and wanting it to win as well as hoping that one’s nation will be a good host are not activities that generate the same lasting sense of impact in terms of increased collective efficacy. Nevertheless, experiences of being together with ethno-racial groups wanting the same things was experienced by many South Africans who attended public view as a unity that could potentially have national impact. This was linked by some participants to previous group experiences of heightened collective emotion at moments of social change (e.g., celebrations surrounding the release of Nelson Mandela in 1990). Despite evidence of the influence of a wide range of top–down imposed and “external” images, messages and practices that created support for the South African team that were then mobilized and felt to spontaneously emerge when the tournament began, it is the normative features of dispositions and the situational emergence (i.e., as affectively realized in parades and Fan Fests) that were emphasized in this analysis rather than a focus on potential causal mechanisms (e.g., of group identification, contagion, etc.). Indeed, the embedded micro and meso-level processes that might be called bottom-up (e.g., sharing with others, dancing in celebration to sustain excitement and express euphoria) and top–down (e.g., billboard messages, advertising, media reports, leader statements) processes could extended in future theorizing with a horizontal ontology (van Dijk and Withagen, 2014) of embedded practices in overlapping contexts and a theory of diachronic group disposition creation and eventual memorialization of the events of the World Cup (e.g., to celebrate instances of national success as realized in the “spontaneous,” intense but not unruly celebrations of the first South African goal and their potential subsequent use as described below). It was evident that the historical nature of the first World Cup on African soil, international media attention and the long, ubiquitous build-up to the event interacted with a largely tacit desire for international recognition and improved national status to create emotions which were experienced during ritual opportunities (i.e., anthem singing) and dependent on game outcomes (e.g., the scoring of a first host nation goal). Both top-down and bottom-up practices reinforced the goals and prior enacted national group commitments necessary for subsequent entrainment during the tournament.

The analysis also reflects the value of the social ontology approach to collective emotions in contrast to versions of social identity theory. The sound of group engagement with a parade or televised game of football and the actions that one engages in with others contribute to an impressive experience in part because such occasions are afforded historical significance and believed by those witnessing them to be special. Rather than explain these emotions in groups as the influence of mechanisms of contagion or a shift mainly in social identity, in this account the willingness of people to take on both the aims of the nation and of a national representative team in ways that may seem personal and “fused” with the group has strong *and* weak effects: strong because intense emotions and feelings of having a shared salient identity can create a sense of unity that replaces previous experiences of multiple group divisions. And weak because the euphoria experienced in a crowd watching a football game may persist and be translated into further beliefs about the nation and collective action only to the extent that it is re-experienced in individual sharing or mobilized in the future in different social and political projects (e.g., to achieve social and political goals). Supporting a national team in virtue of group membership is a limited exercise of agency that is also highly contingent upon factors outside one’s own control (e.g., the performance of a team against other better resourced and more experienced international opponents). In this respect, while collective pride is an appropriate way to understand the times of group emotions about the nations that occur in Fan Fests, it is important to explore similarities and differences with experiences of collective empowerment and the longer term emotional impact of events such as hosting a World Cup: that is, not only explanations that attempt to explain how particular collective emotions are possible until they are realized on particular occasions depending on ritual forms and cultural and historical contingent conditions (e.g., the pride that could emerge given the opportunity for an African team to reach a semi-final in the first African World Cup). Accordingly, mega-sporting events often provide an entertaining but diverting “carnival” that is arguably an opportunity for collective pride and other shared emotions (e.g., group schadenfreude at the decline of a national rival), but the extra-ordinary nature of the event explains why the emotions generated are different from feelings of collective empowerment that motivate groups to pursue collective action to enact social and political change.

There are conceptual and methodological limitations to the research outlined. The sheer scale of some collective emotions means that even case study methods can only approximate events, including how their multiple process unfold and “feed-forward” in complex and often unpredictable ways. Ideally, the interplay between individual, organization, collective, national and transnational features of the emotional topography of an event such as a World Cup should be investigated longitudinally by multiple researchers. Such methodological pluralism would potentially address the limitations of viewing the affects generated by mega-sporting events through the competing lenses of nationalism studies, collective emotion research, social identity theories and social ontology analyses. An important contribution of this case study was to confirm the authoritative role of national and group reasons (e.g., in discussions and discursive-practical representations of national interests) as outlined in social ontology accounts. Nevertheless, approaches and questions were suggested that could be explored in future research such as explanations of the limited spread or sharing in the emotions that occasions of national significance afford and the possibilities for better capturing the affective impact and consequences of enacting national interests in relation to international events. In this study, collective pride helped to organize accounts of group euphoria and excitement as well as feelings (rather than mere perceptions or appraisals) of unity and solidarity, but appropriately refined concepts of embodiment and collective action tendencies should be incorporated in future social identity and social ontology-based studies of collective emotion (e.g., why do particular crowd arrangements and enactments of positive emotion create the embodied “goose flesh” reaction when first experienced and often upon recollection). This should also address the potentialities and topographies of emotion generating and mobilizing group activities and national projects as well as contribute to further understanding of the enduring impact and importance for groups of such peak collective emotional occurrences.

Overall, the future of research on collective emotions in contexts of group celebration, competition and conflict looks especially bright if the insights of new frameworks such as the social ontology and affective practices approaches can ground interdisciplinary integration and examination of previously isolated research programs.

## Author Contributions

GS conducted the research, analyzed the results, and wrote the manuscript.

## Conflict of Interest Statement

The author declares that the research was conducted in the absence of any commercial or financial relationships that could be construed as a potential conflict of interest.
